# Addressing the risk and management of cardiometabolic complications in prostate cancer patients on androgen deprivation therapy and androgen receptor axis-targeted therapy: consensus statements from the Hong Kong Urological Association and the Hong Kong Society of Uro-Oncology

**DOI:** 10.3389/fonc.2024.1345322

**Published:** 2024-01-31

**Authors:** Darren M. C. Poon, Guang-Ming Tan, Kuen Chan, Marco T. Y. Chan, Tim-Wai Chan, Raymond W. M. Kan, Martin H. C. Lam, Clarence L. H. Leung, Kenneth C. W. Wong, Kevin K. H. Kam, Chi-Fai Ng, Peter K. F. Chiu

**Affiliations:** ^1^ Department of Clinical Oncology, State Key Laboratory of Translational Oncology, Sir YK Pao Centre for Cancer, Hong Kong Cancer Institute, The Chinese University of Hong Kong, Hong Kong, Hong Kong SAR, China; ^2^ Comprehensive Oncology Centre, Hong Kong Sanatorium and Hospital, Hong Kong, Hong Kong SAR, China; ^3^ Division of Cardiology, Department of Medicine and Therapeutics, The Chinese University of Hong Kong, Hong Kong, Hong Kong SAR, China; ^4^ Department of Clinical Oncology, Pamela Youde Nethersole Eastern Hospital, Hong Kong, Hong Kong SAR, China; ^5^ Division of Urology, Department of Surgery, Tuen Mun Hospital, Hong Kong, Hong Kong SAR, China; ^6^ Department of Clinical Oncology, Queen Elizabeth Hospital, Hong Kong, Hong Kong SAR, China; ^7^ eFLO Urology, Hong Kong, Hong Kong SAR, China; ^8^ Hong Kong Integrated Oncology Centre, Hong Kong, Hong Kong SAR, China; ^9^ Department of Surgery, Kwong Wah Hospital, Hong Kong, Hong Kong SAR, China; ^10^ Department of Clinical Oncology, The Chinese University of Hong Kong, Hong Kong, Hong Kong SAR, China; ^11^ S.H. Ho Urology Centre, Department of Surgery, The Chinese University of Hong Kong, Hong Kong, Hong Kong SAR, China

**Keywords:** abiraterone, apalutamide, darolutamide, degarelix, enzalutamide, gonadotropin-releasing hormone, leuprolide, luteinizing hormone-releasing hormone

## Abstract

**Background:**

Androgen deprivation therapy (ADT) is the foundational treatment for metastatic prostate cancer (PCa). Androgen receptor (AR) axis-targeted therapies are a new standard of care for advanced PCa. Although these agents have significantly improved patient survival, the suppression of testosterone is associated with an increased risk of cardiometabolic syndrome. This highlights the urgency of multidisciplinary efforts to address the cardiometabolic risk of anticancer treatment in men with PCa.

**Methods:**

Two professional organizations invited five urologists, five clinical oncologists, and two cardiologists to form a consensus panel. They reviewed the relevant literature obtained by searching PubMed for the publication period from April 2013 to April 2023, to address three discussion areas: (i) baseline assessment and screening for risk factors in PCa patients before the initiation of ADT and AR axis-targeted therapies; (ii) follow-up and management of cardiometabolic complications; and (iii) selection of ADT agents among high-risk patients. The panel convened four meetings to discuss and draft consensus statements using a modified Delphi method. Each drafted statement was anonymously voted on by every panelist.

**Results:**

The panel reached a consensus on 18 statements based on recent evidence and expert insights.

**Conclusion:**

These consensus statements serve as a practical recommendation for clinicians in Hong Kong, and possibly the Asia-Pacific region, in the management of cardiometabolic toxicities of ADT or AR axis-targeted therapies in men with PCa.

## 1 Introduction

Despite advances in the understanding of cancer biology and the development of novel medications, the inhibition of plasma testosterone by surgical or pharmacological androgen deprivation therapy (ADT) remains the foundation of systemic treatment for prostate cancer (PCa) (1). Conventional pharmacological ADTs include luteinizing hormone-releasing hormone (LHRH) or gonadotropin-releasing hormone (GnRH) agonists (e.g. leuprolide) and LHRH (or GnRH) antagonists (e.g. degarelix). In recent years, androgen receptor (AR) axis-targeted therapies, such as abiraterone and enzalutamide, have been approved for the treatment of advanced PCa (1). These agents inhibit the biological effects of testosterone by suppressing androgen signaling. However, treatments that target androgen deprivation are reportedly associated with cardiovascular (CV) and metabolic adverse events (AEs) (2).

Because patients with PCa live longer nowadays thanks to advances in therapy, consideration of the potential CV risk of ADT and AR axis-targeted therapy is essential for optimizing patient care in the long term. This notion aligns with the development of cardio-oncology, an emerging subspecialty aimed at improving outcomes for patients on anticancer treatment with potential cardiotoxicity (3). With the expanding older population and the growing burdens of PCa and CV diseases (CVDs) in the Asia-Pacific region (4, 5), the awareness of cardio-oncology should be promoted among clinicians. Between April and September 2023, the Hong Kong Urological Association and the Hong Kong Society of Uro-Oncology convened a series of meetings to discuss and establish consensus statements on the assessment, monitoring, and management of cardiometabolic complications associated with ADT and AR axis-targeted therapies in patients with PCa.

## 2 Methods

The two professional organizations invited several experts to form a consensus panel. They included five urologists, five clinical oncologists, and two cardiologists, all with more than 10 years of experience as a specialist practicing in the public or private healthcare sectors. The panel identified the following three main areas for discussion in consensus meetings: (i) baseline assessment and screening for risk factors in PCa patients before the initiation of ADT and AR axis-targeted therapies; (ii) follow-up and management of cardiometabolic complications; and (iii) selection of ADT agents among high-risk patients.

The panel commissioned a medical writing agency to search for relevant literature on the PubMed database using the following keywords: ‘abiraterone’; ‘androgen deprivation therapy’; ‘apalutamide’; ‘cardiometabolic’; ‘cardiovascular’; ‘darolutamide’; ‘enzalutamide’; ‘gonadotropin releasing hormone’; ‘luteinizing hormone-releasing hormone’; ‘metabolic’; ‘monitoring’; ‘prevention’; ‘prostate cancer’; ‘referral’; and ‘risk assessment’. Types of articles included randomized controlled trials (RCTs), observational studies, reviews, and clinical guidelines published in English between April 2013 and April 2023. The medical writing agency initially sorted the relevant papers by screening the titles and abstracts. The panel further scrutinized the full text of each sorted paper.

The panel was divided into three groups containing four panelists each to address the discussion areas by presenting the relevant papers from the literature search and other studies they deemed appropriate, and sharing their clinical experience in multiple meetings, using the modified Delphi method (6). Each group drafted consensus statements for the corresponding area based on meeting proceedings. At the last meeting, the panel discussed, revised, and finalized the consensus statements. Each statement was voted on anonymously by every panelist based on the practicability of the recommendation. A consensus statement was accepted only if ≥ 80% of the panelists chose ‘accept completely’ or ‘accept with some reservation’ from the five options, which also included ‘accept with major reservation’, ‘reject with reservation’, and ‘reject completely’. The agreement threshold of 80% was adopted in line with the common practice of international Delphi consensus studies (7, 8). **Appendix S1** shows full voting records for all accepted and rejected statements.

## 3 Results

A total of 18 consensus statements (**Table 1**) were accepted and the rationale for each is described below.

**Table 1:** All accepted consensus statements.

Discussion area	Subsection	No.	Statement	References
**1. Baseline assessment and screening for risk factors in PCa patients before the initiation of ADT and AR axis-targeted therapies**	1.1. Baseline assessment items	1	The following lifestyle risk factors should be considered at baseline: a. Smoking history b. Alcohol consumption c. BMI	(9, 10)
		2	A history of the following conditions/therapies should be considered at baseline: a. Diabetes mellitus b. Hypertension c. Hyperlipidemia d. CAD (ACS/MI/coronary revascularization) e. Heart failure f. Arrhythmia g. Cerebrovascular disease h. Peripheral arterial disease i. Chronic kidney disease j. Systemic anticancer therapy k. Radiotherapy to the thorax	(11–18)
		3	ECG can be used to screen for existing CVD before initiating ADT or AR axis-targeted agents.	(19–24)
		4	Cardiac imaging tools, e.g. transthoracic echocardiogram or multigated acquisition scan, for CV risk prediction, could be considered in selected patients.	(19–24)
	1.2. CV risk stratification system	5	An individual’s background ASCVD risk can be assessed using the American College of Cardiology ASCVD Risk Estimator.	(25, 26)
		6	Use of the coronary artery calcium score to assist in the risk assessment of individuals who are at intermediate ASCVD risk can be considered.	(27, 28)
	1.3. Duration of ADT and CVD risk	7	In patients who experienced ≥ 2 prior CV events, the CVD risk is the highest in the first 6 months of ADT; closer monitoring of CV risk should be considered in the first 6 months.	(29)
		8	In patients with clinically significant CVD risk, a shorter duration of ADT or intermittent ADT may be considered after balancing the CV risk and oncologic outcomes.	(30–33)
	1.4. Choice of AR axis-targeted therapy and CVD risk	9	The choice of AR axis-targeted agents should be tailor-made based on the CV risk profiles of individual patients.	(19–24)
		10	Before the initiation of AR axis-targeted therapies, agent-specific CV adverse events should be discussed with the patient.	(34–38)
**2. Follow-up and management of cardiometabolic complications**	2.1. Follow-up schedules	11	After the initiation of ADT or AR axis-targeted therapies, regular monitoring of the following cardiometabolic parameters can be considered: a. Blood pressure b. BMI c. HbA1c d. Lipid profile	(34, 39–45)
		12	In men who have 0–1 CV risk factor and no known history of established ASCVD at baseline, the cardiometabolic parameters in Statement 11 can be checked annually after the initiation of ADT or AR axis-targeted therapies.	(43, 46–48)
		13	More frequent (e.g. every 3–6 months) monitoring of the same cardiometabolic parameters can be considered in patients who have ≥ 2 CV risk factors or a history of established ASCVD at baseline.	(48, 49)
	2.2. Measures to reduce the risk of cardiometabolic complications	14	The ABCDE Cardio-Oncology clinical algorithms* can be considered to reduce the risk of cardiometabolic complications associated with ADT or AR axis-targeted therapies.	(48, 50, 51)
		15	Long-term (> 6 months) combined aerobic and resistance training can be recommended for patients on ADT or AR axis-targeted therapies.	(39, 42, 52–60)
	2.3. Management of cardiometabolic complications	16	Patients with abnormal cardiometabolic parameters should be referred to a cardiologist, endocrinologist, or family physician for further management.	(39, 48, 50, 52)
**3. Selection of ADT agents among high-risk patients**		17	For men with pre-existing ASCVD, LHRH antagonists may be the preferred ADT regimen to LHRH agonists.	(47, 61–76)
		18	For men with treatment-emergent ASCVD during ADT, the ADT regimen should be reviewed.	Expert opinion

ACS, acute coronary syndrome; ADT, androgen deprivation therapy; AR, androgen receptor; ASCVD, atherosclerotic cardiovascular disease; BMI, body mass index; CAD, coronary artery disease; CV, cardiovascular; CVD, cardiovascular disease; ECG, electrocardiogram; HbA1c, glycated hemoglobin; LHRH, luteinizing hormone-releasing hormone; MI, myocardial infarction; PCa, prostate cancer.

*The ABCDE strategy comprises: A, awareness of risks of heart disease and aspirin; B, blood pressure; C, cholesterol and cigarette/tobacco cessation; D, diet and weight management, dose of chemotherapy or radiation, and diabetes mellitus prevention/treatment; and E, exercise, electrocardiogram, and echocardiogram.

### 3.1 Part I: Baseline assessment and screening for risk factors in PCa patients before the initiation of ADT and AR axis-targeted therapies

#### 3.1.1 Baseline assessment items

Statement 1: The following lifestyle risk factors should be considered at baseline:

a. Smoking history
b. Alcohol consumption
c. Body mass index (BMI)

Statement 2: A history of the following conditions/therapies should be considered at baseline:

a. Diabetes mellitus
b.Hypertension
c. Hyperlipidemia
d. Coronary artery disease (acute coronary syndrome [ACS]/myocardial infarction [MI]/coronary revascularization).
e. Heart failure
f. Arrhythmia
g. Cerebrovascular disease
h. Peripheral arterial disease (PAD)
i Chronic kidney disease
j. Systemic anticancer therapy
k. Radiotherapy to the thorax

A number of studies of patients with PCa in Asia including Hong Kong have revealed that ADT users have higher risks of heart failure, hypertension, new-onset diabetes mellitus, dyslipidemia, and being overweight, compared with non-ADT users (11–18). The association between ADT and an increased risk of developing cardiometabolic complications involves multiple factors, which include a decrease in the testosterone level, an elevation in follicle-stimulating hormone (FSH), changes in body composition, increased insulin resistance, and endothelial dysfunction (77).

In Hong Kong, patients with PCa are diagnosed at a median age of 71 years (78). The panel noted that, because of the introduction of effective systemic therapies, patients with PCa now live longer and inevitably face the risk of developing cardiometabolic events associated with prolonged exposure to ADT or AR axis-targeted therapies. It is crucial to assess baseline cardiometabolic profiles of patients with PCa before the initiation of these agents, in order to facilitate subsequent follow-up and early detection of abnormalities, and thus early management.

Based on clinical experience and the literature regarding protection against CVD (9, 10), the panel reached a consensus that the aforementioned items, which include undesirable health behaviors, pre-existing risk factors, and relevant medical history, should be checked at baseline before commencing ADT or AR axis-targeted therapies. The panel noted that these items are predictors of heart failure, MI, hypertension, hyperglycemia, dyslipidemia, and all-cause mortality. They added that the onset time and severity of prior CVDs are important considerations. For instance, the risk–benefit ratio of ADT/AR axis-targeted therapies should be carefully assessed among patients who had a recent MI (e.g. within the past 6 months). Another consideration is the treatment history. Extra attention should be given to patients who received cardiotoxic drugs for the treatment of cancers other than PCa, or patients who received radiotherapy to the thorax, which may increase the risk of CVD due to a high radiation dose to the heart. In clinical practice, clinic stewards and assistants, as well as the Electronic Health Record Sharing System, can facilitate the baseline assessment of the above items.

Statement 3: Electrocardiogram (ECG) can be used to screen for existing CVD before initiating ADT or AR axis-targeted agents.

Statement 4: Cardiac imaging tools, e.g. transthoracic echocardiogram or multigated acquisition scan, for CV risk prediction could be considered in selected patients.

Apart from medical history, it is important to check whether a patient has current CVD at the initiation of ADT/AR axis-targeted therapies. ECG is a useful, convenient diagnostic tool for left ventricular hypertrophy, ischemic and arrhythmic changes, MI, and QT prolongation. Other cardiac imaging tools, such as transthoracic echocardiography and multigated acquisition scan, can be considered in patients with multiple CV risk factors at baseline or who are planned to receive AR axis-targeted therapies, for which safety has not been established in patients with a left ventricular ejection fraction of < 50% or New York Heart Association (NYHA) class III or IV heart failure (19–24). The panel discussed that, despite the prognostic value being increasingly recognized, cardiac serum biomarkers, such as troponins and natriuretic peptides, are seldom tested in routine clinical practice.

#### 3.1.2 CV risk stratification system

Statement 5: An individual’s background atherosclerotic CVD (ASCVD) risk can be assessed using the American College of Cardiology ASCVD Risk Estimator.

Statement 6: Use of the coronary artery calcium score to assist in the risk assessment of individuals who are at intermediate ASCVD risk can be considered.

Among a number of systems for CV risk stratification, the ASCVD Risk Estimator has been widely adopted for primary prevention in the clinical setting in Hong Kong. It estimates the 10-year risk of developing an adverse CV event, such as MI, stroke or severe PAD, based on a set of parameters (**Table 2**) (25). The calculation can be done using online software (26). Other risk stratification tools, such as the coronary calcium score, can be considered for further evaluation of a patient’s CV risk (27, 28).

**Table 2:** Components and risk stratification of the Atherosclerotic Cardiovascular Disease (ASCVD) risk estimator (25, 26).

Parameter	Risk category	10-year risk for ASCVD
**• Current age** **• Sex** **• Race** **• Systolic blood pressure** **• Diastolic blood pressure** **• Total cholesterol** **• HDL cholesterol** **• LDL cholesterol** **• History of diabetes mellitus** **• Smoker status** **• Hypertension treatment status** **• Statin treatment status** **• Aspirin therapy status**	Low	< 5%
	Borderline	5–7.4%
	Intermediate	7.5–19.9%
	High	> 20%
	Very high	Not applicable

HDL, high-density lipoprotein; LDL, low-density lipoprotein.

Age and race are risk factors that are particularly relevant in the context of Hong Kong and the region. An age ≥ 75 years (compared with 71 years being the median age of PCa diagnosis in Hong Kong) is considered a prominent CV risk factor (25), especially in people with multiple comorbidities and a high degree of frailty. People of South Asian race appear to have a three- to five-fold increase in the risks of MI and CV mortality when compared to other ethnic groups (79).

The panel noted that patients who have an intermediate or above 10-year risk for ASCVD or a history of ASCVD (e.g. ACS, MI, stable or unstable angina, coronary or other arterial revascularization, stroke, transient ischemic attack, PAD, or aortic aneurysm) should be referred to a cardiologist or an internal medicine physician for further management.

#### 3.1.3 Duration of ADT and CVD risk

Statement 7: In patients who experienced ≥ 2 prior CV events, the CVD risk is highest in the first 6 months of ADT; closer monitoring of CV risk should be considered in the first 6 months.

A retrospective study conducted in Sweden showed that, compared with an age-matched, PCa-free comparison cohort, men with PCa on ADT had an elevated risk of CVD (hazard ratio [HR] 1.21 and 95% confidence interval [CI] 1.18–1.25 for LHRH agonists; HR 1.16 and 95% CI 1.08–1.25 for orchiectomy). In those with two or more CV events (as per the International Classification of Diseases 10^th^ revision: 100–199) pre-treatment, the risk of CVD was highest during the first 6 months of ADT (HRs 1.91 [95% CI 1.66–2.20], 1.60 [95% CI 1.24–2.06], and 1.79 [95% CI 1.16–2.76] for LHRH agonists, antiandrogens, and orchiectomy, respectively) (29). Based on these data, the panel highlighted the importance of closer monitoring of CV risk in the first 6 months of ADT for patients with multiple prior CV events.

Statement 8: In patients with clinically significant CVD risk, a shorter duration of ADT or intermittent ADT may be considered after balancing the CV risk and oncologic outcomes.

The registry data on patients with nonmetastatic PCa in Norway showed that ADT was associated with increased risks of the composite CV outcome and the individual components, i.e. MI, stroke, and heart failure (adjusted HRs 1.13 [95% CI 1.05–1.21], 1.18 [95% CI 1.05–1.32], 1.21 [95% CI 1.06–1.38], and 1.23 [95% CI 1.13–1.35], respectively), especially among patients who had a moderate CV risk (hypertension ± hypercholesterolemia ± diabetes mellitus; no stroke, MI, or heart failure) and received ADT for a longer duration (> 7 months) (30). The authors also highlighted that, in patients at high CV risk (prior stroke ± MI ± heart failure), the association between ADT and the composite CV outcome was strong, but not statistically significant (HR 1.29; 95% CI 0.92–1.82), because of a lack of power due to a significantly lower number of patients (30).

An analysis of the randomized phase III RTOG 94-08 trial of patients with clinically localized PCa (T1b–2b; prostate-specific antigen [PSA] < 20 ng/mL) demonstrated that 4 months of ADT (with an LHRH agonist and an antiandrogen) plus radiotherapy improved overall survival (OS) and disease-specific survival (DSS), with no elevated 10-year risk of CV mortality (unadjusted HR 1.07; 95% CI 0.81–1.42; P = 0.62), compared with radiotherapy alone (31). Consistent findings were observed in patients at low risk of PCa-related death or at high risk of CV death (31).

A population-based study conducted in the U.S. revealed that, in a cohort of men with advanced PCa, intermittent ADT (with a 90-day interval between two treatment sessions) was associated with a lower risk for serious CV events within 5 years of treatment initiation (i.e. acute MI, stroke, or heart failure; HR 0.64; 95% CI 0.53–0.77; P < 0.0001), particularly heart failure (HR 0.62; 95% CI 0.49–0.78; P < 0.0001), compared with continuous ADT (32). Patients with metastatic and nonmetastatic disease at baseline had similar results (32).

However, the difference in survival outcomes between intermittent and continuous ADT remains dubious. In an RCT, men with newly diagnosed metastatic hormone-sensitive PCa (mHSPC; PSA ≥ 5 ng/mL) received ADT (with an LHRH agonist and an antiandrogen) for 7 months; those with PSA ≤ 4 ng/mL were then randomized to receive intermittent or continuous ADT (33). With a median follow-up of 9.8 years, the intermittent and continuous ADT groups had a median survival of 5.1 and 5.8 years, respectively (HR for death with intermittent ADT, 1.10; 90% CI, 0.99–1.23) (33). The authors cautioned that the CI for survival exceeded the upper boundary for noninferiority, implying that a 20% greater risk of death with intermittent ADT cannot be excluded (33).

In summary, shorter-term (e.g. 4–7 months) or intermittent ADT can be considered to reduce the risk of developing MI, stroke, or heart failure, while preserving survival benefits, in patients with nonmetastatic PCa and a moderate (e.g. hypertension ± hypercholesterolemia ± diabetes mellitus) to high (e.g. prior stroke ± MI ± heart failure) CV risk (30–32). In the setting of metastatic disease, intermittent ADT may mitigate the risk of heart failure (32); however, it should be noted that the comparison of intermittent and continuous ADT in terms of survival outcomes remains inconclusive (33).

#### 3.1.4 Choice of AR axis-targeted therapy and CVD risk

Statement 9: The choice of AR axis-targeted agents should be tailor-made based on the CV risk profiles of individual patients.

Patients who had uncontrolled hypertension or recent ASCVD, of different severities and onset times, were excluded from the landmark trials of AR axis-targeted therapies (**Table 3**) (19–24). These exclusion criteria can serve as a basis (using ECG where applicable) for choosing an AR axis-targeted agent for an individual patient at baseline.

**Table 3:** Exclusion criteria for cardiovascular disease in landmark trials of androgen receptor axis-targeted agents.

	Abiraterone (19, 20)	Enzalutamide (21)	Apalutamide (22)	Darolutamide (23, 24)
Uncontrolled hypertension	SBP ≥ 160 mmHg DBP ≥ 95 mmHg	SBP ≥ 170 mmHg DBP ≥ 105 mmHg	SBP ≥ 160 mmHg DBP ≥ 100 mmHg	SBP ≥ 160 mmHg DBP ≥ 100 mmHg
Severe/unstable angina	Past 6 months	Past 3 months	Past 6 months	Past 6 months
Prior MI	Past 6 months	Past 3 months	Past 6 months	Past 6 months
Heart failure	NYHA class II–IV or cardiac ejection fraction < 50% at baseline	NYHA class II–IV	Symptomatic	NYHA class III or IV
Thrombotic event	Past 6 months	Past 3 months	Past 6 months	Not specified
Cardiac arrhythmia requiring therapy	Requiring therapy (including atrial fibrillation)	Past 3 months	Past 6 months	Not specified
Prior TIA/CVA	Not specified	Past 12 months	Past 12 months	Past 6 months
Coronary/peripheral artery bypass graft	Not specified	Not specified	Not specified	Past 6 months

CVA, cerebrovascular accident; DBP, diastolic blood pressure; MI, myocardial infarction; NYHA, New York Heart Association; SBP, systolic blood pressure; TIA, transient ischemic attack.

Statement 10: Before the initiation of AR axis-targeted therapies, agent-specific CV AEs should be discussed with the patient.

Studies revealed that AR axis-targeted therapies had different profiles of CV AEs (34–38). A meta-analysis of three landmark studies conducted in patients with nonmetastatic castration-resistant PCa (nmCRPC) showed that apalutamide, darolutamide, and enzalutamide were associated with significantly increased risks of hypertension (relative risks [RRs] 1.213, 1.452, and 2.150, respectively) and CV events (RRs 4.466, 2.710, and 2.125, respectively) compared with placebo (34).

Real-world data demonstrated that, in the setting of metastatic CRPC (mCRPC), abiraterone was associated with a higher risk of hospitalization for heart failure compared with enzalutamide (35, 36). Abiraterone should be used cautiously in patients with NYHA class II–IV heart failure or a baseline cardiac ejection fraction < 50% (19, 20). One observational study also showed that abiraterone was associated with a 31% increased risk of MI or stroke compared with enzalutamide (HR, 1.31; 95% CI, 1.05–1.63; P < 0.01) (37).

In a recent systematic review and meta-analysis of RCTs that included patients who received AR axis-targeted therapies for nmCRPC, mCRPC, and mHSPC, enzalutamide was ranked the most toxic regarding hypertension in the settings of nmCRPC and mCRPC (38).

Taken together, the evidence suggests that the risk of hospitalization for heart failure and hypertension associated with abiraterone and enzalutamide, respectively, should be discussed with patients before treatment initiation. The CV side-effect profiles of apalutamide and darolutamide are worth further investigation, as they have recently been approved for the treatment of mHSPC in addition to nmCRPC.

### 3.2 Part II: Follow-up and management of cardiometabolic complications

#### 3.2.1 Follow-up schedules

Statement 11: After the initiation of ADT or AR axis-targeted therapies, regular monitoring of the following cardiometabolic parameters can be considered:

- Blood pressure (BP)
- BMI
- Glycated hemoglobin (HbA1c)
- Lipid profile

ADT or AR axis-targeted therapy may be associated with changes in multiple metabolic parameters over time, possibly through the suppression of testosterone (39). Although the clinical effects of ADT on BP remain inconclusive (39), a systematic review of studies on men with hypogonadism indicated that there was a correlation between low testosterone levels and the development of hypertension (40). With respect to AR axis-targeted agents, an increased risk of hypertension was observed across placebo-controlled RCTs (34). Therefore, monitoring of BP should be considered in patients receiving ADT ± AR axis-targeted therapy.

A retrospective study showed that ADT with LHRH agonists for 48 weeks was associated with increases in body weight (+2.4% ± 0.8%; P < 0.001) and percentage fat body mass (+9.4% ± 1.7%; P < 0.001) in men with nonmetastatic PCa (41). The fat accumulation was mainly subcutaneous rather than intra-abdominal (41). Considering the potential for weight gain and thus increased BMI, which are established CV risk factors, the panel suggested regular monitoring of these parameters in men receiving ADT.

Multiple large cohort studies demonstrated that patients who received ADT for local or locoregional PCa had a 40–60% increased risk of developing diabetes mellitus (39, 42). One prospective cohort study revealed that continuous ADT for 2 years was associated with an elevated level of HbA1c (+0.13% ± 0.34%; P = 0.019) in men without pre-existing diabetes mellitus (43). In a propensity-matched cohort study of PCa men with pre-existing diabetes mellitus who had or had not received ADT, the ADT group had an increase in the mean HbA1c level from baseline to year 1 (+0.14 mmol/L vs. –0.10 mmol/L; P = 0.008) and a higher risk of addition of antidiabetic medication (adjusted HR 1.20; 95% CI 1.09–1.32), compared with the no-ADT group (44). Considering the potential impact of ADT on glycemic control and the development of diabetes mellitus, the panel recognized a need for regular measurements of HbA1c among men on ADT.

Despite results being inconsistent, a number of studies showed that ADT was associated with increased levels of triglycerides, total cholesterol, and low-density lipoprotein cholesterol (39, 42). Dyslipidemia is one component of metabolic syndrome as per the World Health Organization (45). The panel agreed that monitoring of lipid profiles should be considered among patients receiving ADT.

Statement 12: In men who have 0–1 CV risk factor and no known history of established ASCVD at baseline, the cardiometabolic parameters in Statement 11 can be checked annually after the initiation of ADT or AR axis-targeted therapies.

In a guideline developed by experts from Australia on the management of bone and metabolic health in patients on ADT for nonmetastatic PCa, half-yearly to yearly metabolic assessment during the first 2 years of ADT was recommended (46). Recently, the European Society of Cardiology (ESC) and several other associations have also recommended annual CV risk assessment during ADT (47). Several panelists noted that, in public healthcare institutions in Hong Kong, annual blood testing is a routine practice in the follow-up of patients with chronic conditions, such as hypertension, diabetes mellitus, and hypercholesterolemia. The panel considered that it is appropriate and practical to perform yearly monitoring of the cardiometabolic parameters stated in Statement 11 among PCa patients with a low CV risk (i.e. 0–1 CV risk factor and no known history of established ASCVD) at baseline after the start of ADT or AR axis-targeted therapy. CV risk factors include older age (> 60 or > 70 years as per different studies) (43, 47), hypertension, diabetes mellitus, dyslipidemia, cigarette use, family history of CVDs, and components of the metabolic syndrome: (i) abdominal obesity (waist circumference > 40 inches); (ii) triglyceride level ≥ 150 mg/dL; (iii) high-density lipoprotein cholesterol < 40 mg/dL; (iv) systolic BP ≥ 130 mmHg or diastolic BP ≥ 85 mmHg; and (v) fasting glucose ≥ 5.6 mmol/L) (48).

Statement 13: More frequent (e.g. every 3–6 months) monitoring of the same cardiometabolic parameters can be considered in patients who have ≥ 2 CV risk factors or a history of established ASCVD at baseline.

According to a scientific statement from the American Heart Association (48), checking of cardiometabolic parameters every 3 months is recommended for PCa patients with a higher CV risk (i.e. having a history of ASCVD or ≥ 2 CV risk factors, as aforementioned). Several panelists shared that, in routine clinical practice, patients on ADT for PCa are assessed for PSA levels every 6 months; therefore, the cardiometabolic parameters can be checked at the same frequency. The panel discussed that, in patients with a higher CV risk, the cardiometabolic parameters can be checked half-yearly or more frequently (e.g. quarterly), depending on the availability of resources.

Recently, a retrospective cohort study of 45,059 men (~80% with hypertension and ~60% with hypercholesterolemia) treated with ADT (LHRH agonists or antagonists) for PCa showed that the rate of major adverse CV events (MACEs) was lower in the first year (3.9%) than subsequent years (e.g. 19.6% at year 4) after ADT initiation (49). A similar trend was observed for all-cause mortality, which accounted for 65% and 79% of MACEs at year 1 and year 4, respectively, after ADT initiation (49). These results suggest that patients at high CV risk should remain alert and require frequent monitoring (quarterly or half-yearly) beyond 1 year of ADT.

#### 3.2.2 Measures to reduce the risk of cardiometabolic complications

Statement 14: The ABCDE Cardio-Oncology clinical algorithms can be considered to reduce the risk of cardiometabolic complications associated with ADT or AR axis-targeted therapies.

The ABCDE strategy stands for: A, awareness of risks of heart disease and aspirin; B, BP; C, cholesterol and cigarette/tobacco cessation; D, diet and weight management, dose of chemotherapy or radiation, and diabetes mellitus prevention/treatment; and E, exercise, ECG, and echocardiogram. This approach has been widely recommended by international guidelines in cardio-oncology for preventing CVDs and related risk factors in PCa patients on ADT (48, 50, 51).

Statement 15: Long-term (> 6 months) combined aerobic and resistance training can be recommended for patients on ADT or AR axis-targeted therapies.

The panel noted that, despite several limitations, such as small numbers of participants (28–47, 79), heterogeneous outcomes, and varied endpoint parameters (which might be subject to selection bias), a number of studies revealed that regular physical activity involving aerobic and resistance training is effective in improving cardiorespiratory fitness in PCa patients on ADT (39, 52–58). In Australia, users of the LHRH agonist leuprolide are entitled to a discounted exercise program that has demonstrated efficacy in preserving the participants’ body habitus, fitness, and muscle strength (42, 59). One recent meta-analysis and systematic review showed that improvements in exercise tolerance, diastolic BP, lean mass, fat percentage, and biochemical markers, such as fasting glucose and C-reactive protein, were observed in patients on ADT who performed 2–4 exercise sessions, in the form of combined aerobic and resistance training with 60–80% heart rate reserve intensity, per week for 26–40 weeks (60). Based on the current evidence, the panel agreed that long-term (i.e. > 6 months) combined aerobic and resistance training can serve as a preventive measure against cardiometabolic complications associated with ADT or AR axis-targeted therapy.

Although associations between diet and cardiometabolic diseases have been well documented, the panel was unable to find adequate high-level evidence on the clinical benefits and nutritional details necessary to inform a dietary recommendation for PCa patients on ADT or AR axis-targeted therapies, and thus did not establish any consensus statements in this area. However, as highlighted in the ABCDE strategy, diet modification should be one component of a comprehensive management plan for the primary and secondary prevention of CVDs in patients with PCa.

#### 3.2.3 Management of cardiometabolic complications

Statement 16: Patients with abnormal cardiometabolic parameters should be referred to a cardiologist, endocrinologist, or family physician for further management.

Clinical guidelines generally recommend that men who develop cardiometabolic complications during ADT can be treated with statins, antihypertensive agents, antidiabetic drugs (commonly metformin) and aspirin, where applicable (39, 50, 52). Several panelists also discussed the potential antitumor activity of metformin. Two prospective trials revealed that metformin, combined with docetaxel and abiraterone, had no survival benefits compared with placebo in patients with mCRPC (80, 81). Nevertheless, arms A and B of the ongoing STAMPEDE study are still investigating whether adding metformin to the current standard of care for non-diabetic men with mCRPC can reduce the risk of all-cause mortality.

To optimize both oncologic and cardiometabolic outcomes in PCa patients, multidisciplinary input on patient care at ADT initiation and during long-term follow-up is crucial (48). Several panelists noted that treating physicians may consider prescribing the above-mentioned standard therapies for patients who experience cardiometabolic side effects during ADT. However, most of the panel agreed that early referral to corresponding specialists can optimize patient care.

### 3.3 Part III: Selection of ADT agents among high-risk patients

Statement 17: For men with pre-existing ASCVD, LHRH antagonists may be the preferred ADT regimen to LHRH agonists.

Statement 18: For men with treatment-emergent ASCVD during ADT, the ADT regimen should be reviewed.

LHRH agonists and antagonists suppress testosterone levels through different mechanisms (82). LHRH antagonists suppress both luteinizing hormone (LH) and FSH as opposed to LHRH agonists, which primarily suppress LH (61). FSH receptors are found on the luminal endothelial surface of proliferating tissue. They may play a role in the endothelial cell function, fat accumulation, and lipid metabolism that may increase the risks of plaque destabilization/acute rupture and CVD in patients receiving LHRH agonists (61–64). One pre-clinical study in male rats demonstrated that LHRH agonists were associated with more impairment of endothelial function in the aorta and intrarenal arteries compared with LHRH antagonists (65). However, a phase II randomized open-label study revealed that patients with advanced PCa and pre-existing CVD who received LHRH agonists or antagonists for 1 year had no difference in endothelial function. In contrast, those on LHRH antagonists had an 18.1% absolute risk reduction of a major CV or cerebrovascular AE versus those on agonists (66).

Indeed, results from clinical research that compared the CV safety of LHRH agonists and antagonists have remained mixed. Several retrospective studies conducted in European countries and the U.S. revealed that these two types of ADT had similar overall CV side-effect profiles (67, 83–85). However, one study using the pharmacovigilance information captured in the Eudra-Vigilance and Food and Drug Administration databases showed that, regarding specific CV disorders, the LHRH antagonist degarelix presented lower risks of hypertension (pooled relative risk [PRR] 0.60; 95% CI 0.37–0.98; P = 0.04) and MI (PRR 0.05; 95% CI 0.01–0.39; P < 0.01) compared with LHRH agonists (67).

Two observational studies on Asian patients (68, 69) and one analysis of RCTs (61) demonstrated that LHRH antagonists offered more favorable CV outcomes. A population-based study in Taiwan showed that LHRH antagonists were associated with a lower risk of composite CV events (ischemic heart disease, stroke, congestive heart failure, or CV death) compared with LHRH agonists across different patient subgroups, which included those with metastatic disease (adjusted HR 0.16; 95% CI 0.04–0.38; P = 0.013), those receiving ADT for > 6 months (adjusted HR 0.30; 95% CI 0.16–0.54; P < 0.0001), and those with pre-existing CVD (adjusted HR 0.16; 95% CI 0.05–0.50; P = 0.0017) (68). Another cohort study in Taiwan yielded similar outcomes, suggesting that the effects of LHRH agonists on macrophages may contribute to a higher risk of CV events relative to LHRH antagonists (69).

In a *post hoc* analysis of six phase III RCTs of patients with pre-existing CVD who received ADT for 12 months mostly, the LHRH antagonist degarelix was associated with a significantly lower risk of cardiac events (arterial embolic/thrombotic events, hemorrhagic/ischemic cerebrovascular conditions, MI, and other ischemic heart disease) or death compared with LHRH agonists within 1 year of treatment (HR 0.44; 95% CI 0.26–0.74; P = 0.002) (61).

In recent years, two landmark RCTs provided new insights into the CV risk of LHRH antagonists versus agonists (70, 71). In the phase III HERO study (70), patients with advanced PCa (> 90% of whom had ≥ 1 CV risk factor, e.g. diabetes mellitus, hypertension, or a history of a MACE) were randomized to receive relugolix (an oral LHRH antagonist; N = 622) or leuprolide (an LHRH agonist; N = 308). The primary endpoint showed that relugolix was associated with a significantly higher rate of sustained testosterone suppression (castration levels < 50 ng/dL; 96.7% vs. 88.8%; P < 0.001) compared with leuprolide through 48 weeks. Regarding safety, relugolix was associated with a significantly lower incidence of MACEs (including nonfatal MI, nonfatal stroke, and all-cause mortality) compared with leuprolide (2.9% vs. 6.2%; HR 0.46; 95% CI 0.24–0.88; P < 0.001) (70). Among patients with a prior MACE, there was a more marked difference in the incidences of MACEs between the two treatment arms (3.6% for relugolix vs. 17.8% for leuprolide) (70). Notably, MACE outcomes were the secondary endpoint of the HERO study (70).

In another landmark trial, PRONOUNCE, patients with PCa and pre-existing ASCVD were randomized to receive the LHRH antagonist degarelix (N = 276) or the LHRH agonist leuprolide (N = 269), with the time to first adjudicated MACE (composite of death, MI, or stroke) through 1 year being the primary endpoint (71). However, the study was terminated prematurely because of the smaller-than-planned number of participants and primary outcome events, and the two treatment groups had no significant difference in the incidences of MACEs at 1 year (5.5% for degarelix vs. 4.1% for leuprolide; HR 1.28; 95% CI 0.59–2.79; P = 0.53) (71). As expected, testosterone levels, rates of progressive disease, and urinary symptoms were comparable between the two groups (71).

In contrast to HERO, PRONOUNCE demonstrated that patients treated with degarelix appeared to have a trend towards an increased risk of MACEs; however, the PRONOUNCE investigators emphasized that the results were inconclusive because of wide CIs and low statistical power (71). They also stated that, when using an AE definition similar to that of HERO, degarelix was associated with numerically fewer MACEs than leuprolide, although the difference remained not statistically significant (71).

In response to the inconsistent results from these two RCTs, Ng et al. commented that the intense input and close monitoring by cardiologists in the design of PRONOUNCE might facilitate earlier intervention for CV risk factors and, therefore, reduce the incidence of MACEs in the LHRH agonist group, as compared to their counterparts in HERO (4.1% vs. 17.8%) (72). They also suggested that the association between the intensity of CV interventions at baseline and the CV risk of leuprolide in PRONOUNCE could be further investigated (72).

Recent meta-analyses and systematic reviews of RCTs indicated that LHRH antagonists were associated with fewer CV events, with PRRs ranging from 0.5 to 0.6, compared with LHRH agonists (73–75). However, the expert panel pointed out several caveats. First, the overall number of study participants on LHRH antagonists was much lower than that on LHRH agonists. Second, all studies assessed CV events as a safety outcome or secondary endpoint. Third, durations of ADT in the included studies were relatively short (mostly ≤ 12 months). Fourth, baseline patient characteristics regarding CV risk factors and history of CVD were heterogeneous across the studies. Finally, data on Asian patients were limited.

In summary, no high-level evidence is available to distinguish the CV safety of LHRH agonists and antagonists. Despite a number of confounders and limitations, multiple real-world non-randomized studies and pooled data from RCTs demonstrated that LHRH antagonists were associated with a lower risk of CV events compared with LHRH agonists, especially in PCa patients with pre-existing CVD (61, 67–69, 73–75). Importantly, no prospective data suggested that LHRH antagonists are associated with a higher CV risk than LHRH agonists. Results from the recent landmark study PRONOUNCE implied that optimal CV interventions might alleviate the CV risk of LHRH agonists; however, further investigations are warranted to confirm this hypothesis (72). Notably, a retrospective cohort study conducted in Hong Kong discovered that a longer duration of LHRH agonist therapy was associated with a higher risk of the composite endpoint of MI and stroke (sub-HR per year 1.04; 95% CI 1.01–1.06; P = 0.001), with a more prominent effect observed for a treatment duration of ≥ 2 years (sub-HR 1.23; 95% CI 1.04–1.46; P = 0.017) (76).

In line with the recent ESC guidelines on cardio-oncology (47), the expert panel achieved a consensus that LHRH antagonists may be preferred over LHRH agonists as the ADT regimen for men with pre-existing ASCVD. Additionally, they agreed that the ADT regimen, including the type of the agent and treatment duration, should be reviewed when patients develop suspected ADT-emergent ASCVD.

## 4 Discussion

This set of consensus statements aimed to optimize the care for patients treated with ADT or AR axis-targeted therapies for PCa. Although these agents have significantly improved patient survival, the suppression of testosterone is associated with an increased risk of cardiometabolic syndrome. With an ageing population worldwide, both PCa and cardiometabolic diseases are expected to rise in the coming years. This highlights the urgency of multidisciplinary efforts to address the cardiometabolic risk of anticancer treatment in men with PCa. However, relevant guidelines and recommendations are limited in the Asia-Pacific region. This expert panel anticipated that the consensus statements herein would facilitate the management of CVDs and metabolic syndrome associated with ADT and AR axis-targeted therapies by emphasizing the baseline assessment and follow-up monitoring of risk factors and introducing mitigation measures.

Several caveats should be noted. First, the level of evidence of most studies included in this consensus was low to moderate, because of retrospective study designs, small numbers of participants, and relatively short durations of follow-up. Second, there were heterogeneities in baseline patient characteristics and outcome measures across these studies. Third, with respect to the comparison of LHRH agonists and antagonists, no conclusive data are available, especially among Asian patients. This field of interest should be investigated in RCTs with CV outcomes (e.g. MI, stroke, heart failure, and CV mortality) as primary endpoints, adequately powered statistical plans, and balanced baseline patient characteristics regarding prior CVDs and CV risk factors between treatment arms. Fourth, the potential for cardiotoxicity of newer therapies for mCRPC, such as poly-ADP-ribose polymerase inhibitors, should be further assessed and monitored (86, 87).

Despite these limitations, the consensus statements were formulated based on the recent evidence, supplemented by the insights and expertise of the panelists. These serve as a practical recommendation for clinicians in Hong Kong and possibly the Asia-Pacific region involved in managing cardiometabolic safety of ADT or AR axis-targeted therapies in men with PCa.

## 5 Conclusion

A multidisciplinary panel of clinical oncologists, urologists, and cardiologists with extensive clinical experience in Hong Kong established 18 consensus statements to address the cardiometabolic complications associated with ADT or AR axis-targeted therapies in patients with PCa. Depending on future research findings, these statements are subject to regular review and necessary updating.

## Author contributions

DP: Conceptualization, Project administration, Supervision, Writing – original draft, Writing – review & editing, Data curation, Formal Analysis. G-MT: Writing – review & editing, Data curation, Formal Analysis. KC: Writing – review & editing, Data curation, Formal Analysis. MC: Writing – review & editing, Data curation, Formal Analysis. T-WC: Writing – review & editing, Data curation, Formal Analysis. RK: Writing – review & editing, Data curation, Formal Analysis. ML: Writing – review & editing, Data curation, Formal Analysis. CL: Writing – review & editing, Data curation, Formal Analysis. KW: Writing – review & editing, Data curation, Formal Analysis. KK: Writing – review & editing, Data curation, Formal Analysis. C-FN: Writing – review & editing, Data curation, Formal Analysis. PC: Conceptualization, Data curation, Formal Analysis, Project administration, Supervision, Writing – original draft, Writing – review & editing.

## Supplementary material

Appendix S1 Full voting records for all accepted and rejected statements.
