# Supplementary material for: Addressing the risk and management of cardiometabolic complications in prostate cancer patients on androgen deprivation therapy and androgen receptor axis-targeted therapy: consensus statements from the Hong Kong Urological Association and the Hong Kong Society of Uro-Oncology
Source: Front Oncol. 2024 Jan 31;14:1345322. doi: 10.3389/fonc.2024.1345322 (PMC10864500; doi:10.3389/fonc.2024.1345322)
Supplement: Appendix S1 — Full voting records for all accepted and rejected statements. [file Table_1.docx]

Supplementary Material

**Appendix S1. Full voting records for all accepted and rejected statements.**

**Part 1 – Baseline assessment and screening for risk factors before the initiation of androgen deprivation therapy (ADT) and androgen receptor (AR) axis-targeted therapies**

| **1.1. Baseline assessment items** | | **Response options* (%)** | | | |  | **A statement was accepted only if (A + B)% ≥ 80%** | | |
| --- | --- | --- | --- | --- | --- | --- | --- | --- | --- |
| **Statement #** | **Drafted statements** | **A** | **B** | **C** | **D** | **E** | **A + B (%)** | **Accepted** | **Voted down** |
| 1a | The following lifestyle risk factors should be considered at baseline: Smoking history | 100 | 0 | 0 | 0 | 0 | 100 | ✓ |  |
| 1b | The following lifestyle risk factors should be considered at baseline: Alcohol consumption | 67 | 33 | 0 | 0 | 0 | 100 | ✓ |  |
| 1c | The following lifestyle risk factors should be considered at baseline: Body mass index (BMI) | 100 | 0 | 0 | 0 | 0 | 100 | ✓ |  |
| 1d | The following lifestyle risk factors should be considered at baseline: Waist circumference | 17 | 58 | 17 | 8 | 0 | 75 |  | X |
| 2a | A history of the following conditions / therapies should be considered at baseline: Diabetes mellitus | 100 | 0 | 0 | 0 | 0 | 100 | ✓ |  |
| 2b | A history of the following conditions / therapies should be considered at baseline: Hypertension | 100 | 0 | 0 | 0 | 0 | 100 | ✓ |  |
| 2c | A history of the following conditions / therapies should be considered at baseline: Hyperlipidemia | 100 | 0 | 0 | 0 | 0 | 100 | ✓ |  |
| 2d | A history of the following conditions / therapies should be considered at baseline: Coronary artery disease (acute coronary syndrome/myocardial infarction/coronary revascularization) | 100 | 0 | 0 | 0 | 0 | 100 | ✓ |  |
| 2e | A history of the following conditions / therapies should be considered at baseline: Heart failure | 83 | 17 | 0 | 0 | 0 | 100 | ✓ |  |
| 2f | A history of the following conditions / therapies should be considered at baseline: Arrhythmia | 67 | 33 | 0 | 0 | 0 | 100 | ✓ |  |
| 2g | A history of the following conditions / therapies should be considered at baseline: Cerebrovascular disease | 100 | 0 | 0 | 0 | 0 | 100 | ✓ |  |
| 2h | A history of the following conditions / therapies should be considered at baseline: Peripheral arterial disease | 83 | 8 | 8 | 0 | 0 | 92 | ✓ |  |
| 2i | A history of the following conditions / therapies should be considered at baseline: Chronic kidney disease | 67 | 33 | 0 | 0 | 0 | 100 | ✓ |  |
| 2j | A history of the following conditions / therapies should be considered at baseline: Systemic anticancer therapy | 92 | 8 | 0 | 0 | 0 | 100 | ✓ |  |
| 2k | A history of the following conditions / therapies should be considered at baseline: Radiotherapy to the thorax | 75 | 17 | 8 | 0 | 0 | 92 | ✓ |  |
| 3 | Electrocardiogram can be used to screen for existing cardiovascular (CV) disease (CVD) before initiating ADT or AR axis-targeted agents. | 67 | 33 | 0 | 0 | 0 | 100 | ✓ |  |
| 4 | Cardiac imaging tools, e.g. transthoracic echocardiogram or multigated acquisition scan, for CV risk prediction, could be considered in selected patients. | 42 | 42 | 8 | 8 | 0 | 84 | ✓ |  |
|  |  |  |  |  |  |  | **Total:** | 16 | 1 |

*Response options include A: accept completely; B: accept with some reservation; C: accept with major reservation; D: reject with reservation; and E: reject completely.

| **1.2. CV risk stratification system** | | **Response options* (%)** | | | |  | **A statement was accepted only if (A + B)% ≥ 80%** | | |
| --- | --- | --- | --- | --- | --- | --- | --- | --- | --- |
| **Statement #** | **Drafted statements** | **A** | **B** | **C** | **D** | **E** | **A + B (%)** | **Accepted** | **Voted down** |
| 5 | An individual’s background atherosclerotic CVD (ASCVD) risk can be assessed using the American College of Cardiology ASCVD Risk Estimator. | 83 | 17 | 0 | 0 | 0 | 100 | ✓ |  |
| 6 | Use of the coronary artery calcium score to assist the risk assessment of individuals who are at intermediate ASCVD risk can be considered. | 50 | 42 | 8 | 0 | 0 | 92 | ✓ |  |
|  |  |  |  |  |  |  | **Total:** | 2 | 0 |

*Response options include A: accept completely; B: accept with some reservation; C: accept with major reservation; D: reject with reservation; and E: reject completely.

| **1.3. Duration of ADT and CVD risk** | | **Response options* (%)** | | | |  | **A statement was accepted only if (A + B)% ≥ 80%** | | |
| --- | --- | --- | --- | --- | --- | --- | --- | --- | --- |
| **Statement #** | **Drafted statements** | **A** | **B** | **C** | **D** | **E** | **A + B (%)** | **Accepted** | **Voted down** |
| 7 | In patients who experienced ≥ 2 prior CV events, the CVD risk is the highest in the first 6 months of ADT; closer monitoring of CV risk should be considered in the first 6 months. | 100 | 0 | 0 | 0 | 0 | 100 | ✓ |  |
| 8 | In patients with clinically significant CVD risk, a shorter duration of ADT or intermittent ADT may be considered after balancing the CV risk and oncologic outcomes. | 75 | 25 | 0 | 0 | 0 | 100 | ✓ |  |
|  |  |  |  |  |  |  | **Total:** | 2 | 0 |

*Response options include A: accept completely; B: accept with some reservation; C: accept with major reservation; D: reject with reservation; and E: reject completely.

| **1.4. Choice of AR axis-targeted therapy and CVD risk** | | **Response options* (%)** | | | |  | **A statement was accepted only if (A + B)% ≥ 80%** | | |
| --- | --- | --- | --- | --- | --- | --- | --- | --- | --- |
| **Statement #** | **Drafted statements** | **A** | **B** | **C** | **D** | **E** | **A + B (%)** | **Accepted** | **Voted down** |
| 9 | The choice of AR axis-targeted agents should be tailor-made based on the CV risk profiles of individual patients. | 83 | 17 | 0 | 0 | 0 | 100 | ✓ |  |
| 10 | Before the initiation of AR axis-targeted therapies, agent-specific CV adverse events should be discussed with the patient. | 100 | 0 | 0 | 0 | 0 | 100 | ✓ |  |
|  |  |  |  |  |  |  | **Total:** | 2 | 0 |

*Response options include A: accept completely; B: accept with some reservation; C: accept with major reservation; D: reject with reservation; and E: reject completely.

**Part 2 – Follow-up and management of cardiometabolic complications after the initiation of ADT or AR axis-targeted therapies**

| **2.1. Follow-up schedules** | | **Response options* (%)** | | | |  | **A statement was accepted only if (A + B)% ≥ 80%** | | |
| --- | --- | --- | --- | --- | --- | --- | --- | --- | --- |
| **Statement #** | **Drafted statements** | **A** | **B** | **C** | **D** | **E** | **A + B (%)** | **Accepted** | **Voted down** |
| 11a | After the initiation of ADT or AR axis-targeted therapies, regular monitoring of the following cardiometabolic parameters can be considered: Blood pressure | 100 | 0 | 0 | 0 | 0 | 100 | ✓ |  |
| 11b | After the initiation of ADT or AR axis-targeted therapies, regular monitoring of the following cardiometabolic parameters can be considered: BMI | 75 | 8 | 17 | 0 | 0 | 83 | ✓ |  |
| 11c | After the initiation of ADT or AR axis-targeted therapies, regular monitoring of the following cardiometabolic parameters can be considered: Glycated hemoglobin | 75 | 25 | 0 | 0 | 0 | 100 | ✓ |  |
| 11d | After the initiation of ADT or AR axis-targeted therapies, regular monitoring of the following cardiometabolic parameters can be considered: Lipid profile | 75 | 25 | 0 | 0 | 0 | 100 | ✓ |  |
| 11e | After the initiation of ADT or AR axis-targeted therapies, regular monitoring of the following cardiometabolic parameters can be considered: Waist circumference | 8 | 50 | 25 | 17 | 0 | 58 |  | X |
| 12 | In men who have 0–1 CV risk factor and no known history of established ASCVD at baseline, the cardiometabolic parameters in Statement 11 can be checked annually after the initiation of ADT or AR axis-targeted therapies. | 83 | 17 | 0 | 0 | 0 | 100 | ✓ |  |
| 13 | More frequent (e.g. every 3–6 months) monitoring of the same cardiometabolic parameters can be considered in patients who have ≥ 2 CV risk factors or a history of established ASCVD at baseline. | 75 | 25 | 0 | 0 | 0 | 100 | ✓ |  |
|  |  |  |  |  |  |  | **Total:** | 6 | 1 |

*Response options include A: accept completely; B: accept with some reservation; C: accept with major reservation; D: reject with reservation; and E: reject completely.

| **2.2. Measures to reduce the risk of cardiometabolic complications** | | **Response options* (%)** | | | |  | **A statement was accepted only if (A + B)% ≥ 80%** | | |
| --- | --- | --- | --- | --- | --- | --- | --- | --- | --- |
| **Statement #** | **Drafted statements** | **A** | **B** | **C** | **D** | **E** | **A + B (%)** | **Accepted** | **Voted down** |
| 14 | The ABCDE Cardio-Oncology clinical algorithms can be considered to reduce the risk of cardiometabolic complications associated with ADT or AR axis-targeted therapies. | 67 | 33 | 0 | 0 | 0 | 100 | ✓ |  |
| 15 | Long-term (> 6 months) combined aerobic and resistance training can be recommended for patients on ADT or AR axis-targeted therapies. | 17 | 83 | 0 | 0 | 0 | 100 | ✓ |  |
|  |  |  |  |  |  |  | **Total:** | 2 | 0 |

*Response options include A: accept completely; B: accept with some reservation; C: accept with major reservation; D: reject with reservation; and E: reject completely.

| **2.3. Management of cardiometabolic complications** | | **Response options* (%)** | | | |  | **A statement was accepted only if (A + B)% ≥ 80%** | | |
| --- | --- | --- | --- | --- | --- | --- | --- | --- | --- |
| **Statement #** | **Drafted statements** | **A** | **B** | **C** | **D** | **E** | **A + B (%)** | **Accepted** | **Voted down** |
| 16 | Patients with abnormal cardiometabolic parameters should be referred to a cardiologist, endocrinologist, or family physician for further management. | 100 | 0 | 0 | 0 | 0 | 100 | ✓ |  |
|  |  |  |  |  |  |  | **Total:** | 1 | 0 |

*Response options include A: accept completely; B: accept with some reservation; C: accept with major reservation; D: reject with reservation; and E: reject completely.

**Part 3 – Selection of ADT agents among high-risk patients**

| **Response options* (%)** | | | | | |  | **A statement was accepted only if (A + B)% ≥ 80%** | | |
| --- | --- | --- | --- | --- | --- | --- | --- | --- | --- |
| **Statement #** | **Drafted statements** | **A** | **B** | **C** | **D** | **E** | **A + B (%)** | **Accepted** | **Voted down** |
| 17 | For men with pre-existing ASCVD, luteinizing hormone-releasing hormone (LHRH) antagonists may be the preferred ADT regimen to LHRH agonists. | 67 | 25 | 8 | 0 | 0 | 92 | ✓ |  |
| 18 | For men with treatment-emergent ASCVD during ADT, the ADT regimen should be reviewed. | 83 | 17 | 0 | 0 | 0 | 100 | ✓ |  |
|  |  |  |  |  |  |  | **Total:** | 2 | 0 |

*Response options include A: accept completely; B: accept with some reservation; C: accept with major reservation; D: reject with reservation; and E: reject completely.
